# Impacts of the SARS-CoV-2 Pandemic on the Mental Health of the Elderly

**DOI:** 10.3389/fpsyt.2020.00841

**Published:** 2020-08-17

**Authors:** Wendney Hudson de Alencar Fontes, Jucier Gonçalves Júnior, Carlos Augusto Carvalho de Vasconcelos, Cláudio Gleidiston Lima da Silva, Maria Socorro Vieira Gadelha

**Affiliations:** ^1^Faculdade de Medicina Estácio de Juazeiro do Norte, Estácio de Sá de Vitória College, Juazeiro do Norte, Brazil; ^2^Santa Casa de Misericórdia de Fortaleza, Fortaleza, Brazil; ^3^Department of Nutrition, Federal University of Pernambuco, Recife, Brazil; ^4^Faculty of Medicine, Federal University of Cariri, Ceara, Brazil

**Keywords:** coronavirus infections, mental health, health services for the aged, pandemic, public health, mental health disorders

## Introduction

According to the World Health Organization (WHO), SARS-CoV-2 has infected approximately 17 million people worldwide, and almost 670,000 have died from complications of the disease (1). Hence, countries around the world have implemented social distancing measures to reduce the spread of the virus. Coronavirus coping strategies have profoundly changed social dynamics, given the adverse effects on people’s mental health (2) and their psychosocial impact (3). Due to higher morbidity and mortality (4, 5) and potential previous mental illnesses (6), the elderly population should be given more considerable attention, considering they must adhere more appropriately and for more extended periods to preventive measures (7). However, despite these studies, the psychiatric impact of COVID-19 on the elderly population still lacks more significant theoretical support, since few reports are describing psychiatric symptoms associated with the pandemic (5). Given the above, this paper is intended to illustrate and correlate the mental, psychiatric, and psychological consequences for the elderly during the COVID-19 pandemic.

## Materials and Methods

The authors searched in three electronic databases: PubMed (NCBI), Science Direct, and Google Scholar. They used the following search terms with adjustments and variants:

# 1 “COVID-19” OR “SARS-CoV-2” (Medical Subject Headings – (MeSH term)) AND
# 2 “Elderly people” (keywords) OR “Aged” (MeSH term) AND
# 3 “Mental health” (MeSH term) OR “Mental health disorders” (keywords).

Papers were chosen using the following criteria: at least a combination of two terms described in the search strategy; approach on the psychiatric impact of the COVID-19 pandemic on the elderly and original papers with the full text available. An additional search was also carried out on websites and available documents relevant to the theme but which did not previously fit the search.

## Discussion

### Vulnerability of the Elderly

Older people are among the most vulnerable and high-risk groups during epidemics (7, 8), as they often have associated comorbidities (4). The incidence of systemic hypertension in people who are over 60 years old ranges between 45.5 and 63.1%, that of diabetes mellitus is around 16.8 and 26.8% (9, 10), heart failure accounts for 3.8%, and COPD is found in 23.7% (9, 10). These diseases can potentially affect the prognosis of patients with COVID-19, as there may be damage to vascular structures, impaired lung function, and even reduced immunity (11). Also, the elderly naturally have a relatively less effective immune system than young people and are more susceptible to developing critical illnesses (8, 11). Thus, the elderly population can be considered at high risk of disease progression and death from COVID-19. Therefore, the very notion of vulnerability, previous comorbidities such as heart failure, and chronic obstructive pulmonary disease that increase the risk of depression, anxiety disorders, and functional limitations caused by these comorbidities can be significant stressors for the psychological distress of this population.

Another factor that increases the vulnerability of the elderly is limited access to healthcare services. Although they use it more frequently and have higher rates of hospitalizations compared to younger people (12) due to their cardiovascular comorbidities and cognitive and psychotic disorders (2, 8, 9, 11, 13), according to van Gaans and Dent (14), the elderly still face problems in accessing healthcare services. This problem may be due to uneven geographical and spatial distribution of healthcare services, insufficient availability, and difficulty obtaining information. These aspects contribute to a weakness that can be aggravated by financial and health crises, in which there are cuts and reduced spending on healthcare (14). This, in turn, generates concerns and anxiety about the future (15). Thus, it is reasonable to assume that the pandemic’s economic crisis will also affect the mental health of the elderly while significantly reducing their mental healthcare.

Another essential issue to be outlined is that the elderly are subject to social distancing, affecting their psychological and psychiatric status. Most older people have little means of socializing with other people, some being restricted to community centers and religious temples (16, 17). These places may be inaccessible due to lockdowns, which generates feelings of social and psychological isolation in this age group (16, 17). Also, visits to nursing homes have been restricted or even banned, making interaction with family members more difficult and exacerbating nursing homes’ isolation feelings (18). These are worrying facts because they can be triggers (7) for previous psychiatric disorders (8, 13, 18), as they limit therapeutic adherence, amplify negative symptoms, and reinforce self-destructive tendencies from a view of “I am not necessary (…), I have been forgotten (…), I am alone and lonely.” Considering today’s demographic transition around the world, which points to clear population aging, especially in developing countries where psychiatric care is limited, the elderly’s mental health of the elderly and their vulnerability factors become a concern in global public health (7, 17).

### Mental Disorders in the Elderly During the Pandemic

During the COVID-19 epidemic, lack of interaction and social distancing exacerbate psychological disorders and increase the risk of depression and anxiety in the elderly (16, 17). Meng et al. (8) showed that about 37.1% of the elderly had experienced depression and anxiety during the pandemic. In addition to isolation, fear and stress contribute to the onset and exacerbation of pre-existing mental health disorders. People with obsessive-compulsive disorder have higher chances of experiencing obsessive thoughts due to precautionary measures (19). However, there is a lack of consensus concerning these data. A cross-sectional study carried out in China found that 33% of people show anxiety disorders, and about 20% show depressive symptoms. Still, it argued that these data should be lower in more advanced age groups (20).

A robust predictive factor for psychiatric comorbidities is dementia, which is common with advancing age (2, 18). Subjects with dementia and cognitive impairment have limited access to accurate information and facts about the pandemic (18). Also, they may not correctly follow recommendations to reduce the spread of COVID-19 (such as hygiene and precautionary measures), because they cannot remember procedures or understand important information (3, 18). Social distancing effects are also reflected in people with dementia due to withdrawal from important non-pharmacological therapies to treat comorbidities, such as social activities, physical exercises, and group therapies (2). Possible trauma resulting from these changes can further accelerate cognitive decline. As subjects with dementia are more likely to have cardiovascular disease and diabetes (2), it can be assumed that this group is at an even higher risk of morbidity and mortality from COVID-19.

Like dementia, psychosis requires special attention. Social distancing measures can increase psychotic patients’ stress, just like precautions related to disease spread have been associated with increased paranoia (13). The excess of information can also intensify paranoid symptoms, generating suspicions regarding healthcare (3). In this case, patients with psychosis are less motivated to comply with recommended measures (13), leading them to avoid social distancing and quarantine measures (3). Findings show that COVID-19 has been associated with a 25% increase in the incidence of psychotic outbreaks cases (13, 21). In the elderly, there has also been an increase in the risk of schizophrenia, as the mean age for patients newly diagnosed with schizophrenia changed from 39 to 50 years (21). The severity of symptoms and steroid administration seem to contribute to the onset of psychotic symptoms (12). Similarly, there are reports of recent psychosis in infected individuals, and SARS-CoV-2 may have a neuropathogenic mechanism that would trigger these symptoms (5, 13).

### Suggested Proposals

Because of the crisis caused by the pandemic, intervention and preventive measures must be implemented to mitigate and reduce the risk of psychological impact and psychiatric disorders in the elderly (6, 8, 17, 22), namely:

- Expanding telehealth services for the elderly/their family members to answer questions about symptoms, establishing contact to monitor access/medication administration and suggest non-pharmacological adjuvant therapy (e.g., cognitive-behavioral therapy sessions that can be attended online) (7, 17);
- Using telepsychiatry as a screening tool for cases of elderly people with mild/moderate psychiatric disorders, and an assessment tool for cases requiring hospitalization/strict monitoring, such as psychoses (2, 17);
- Preparing training materials for health professionals based on past experiences to qualify them to provide care and act as multipliers of good mental health practices in the pandemic (7, 21);
- Offering advertisement and educational materials to make people aware of the need to interact/care and respect their elderly relatives, the need to maintain regular contact online/through the telephone (3) during the pandemic, and health promotion measures to fight COVID-19 and mental health disorders (8);
- Introducing social security measures to fight the economic exclusion of these individuals (15, 22).

## Conclusion

With the spread of COVID-19, health authorities, governments, and the civil society must deeply reflect on the issue in an attempt to offer equity of care and formulate emergency public policies to deal with the short and long term psychiatric effects of the pandemic, especially in the most vulnerable groups: children, mental patients, refugees, indigenous people, quilombolas (Afro-Brazilian residents of settlements created by escaped slaves), people with chronic non-communicable diseases and, of course, the elderly.

## Author Contributions

JGJ, WHAF, and CACV designed the review, developed the inclusion criteria, screened titles and abstracts, appraised the quality of included papers, and drafted the manuscript. MSVG, CGLS, and CACV reviewed the methodological protocol and inclusion criteria and provided substantial input to the manuscript. JGJ, WHAF, CACV, CGLS, and MSVG reviewed the study protocol.

## Conflict of Interest

The authors declare that the research was conducted in the absence of any commercial or financial relationships that could be construed as a potential conflict of interest.
